# Trends and characteristics of attempted and completed suicides reported to general practitioners before *vs* during the COVID-19 pandemic in France: Data from a nationwide monitoring system, 2010–2022

**DOI:** 10.1371/journal.pone.0278266

**Published:** 2022-12-15

**Authors:** Marie Pouquet, Titouan Launay, Mathieu Rivière, Christine Chan-Chee, Frédéric Urbain, Nicolas Coulombel, Isabelle Bardoulat, Romain Pons, Caroline Guerrisi, Thierry Blanchon, Thomas Hanslik, Nadia Younes

**Affiliations:** 1 INSERM, Institut Pierre Louis d’Epidémiologie et de Santé Publique, Sorbonne Université, Paris, France; 2 Santé Publique France, Saint Maurice, France; 3 Unité de Formation et de Recherche des Sciences de la Santé Simone-Veil, Université de Versailles Saint-Quentin-en-Yvelines, Versailles, France; 4 Real World Solutions Department, IQVIA, Paris, France; 5 Service de Médecine Interne, Hôpital Ambroise-Paré, Assistance Publique - Hôpitaux de Paris, Boulogne-Billancourt, France; 6 Université Versailles Saint Quentin, Université Paris Saclay, Team DevPsy, Villejuif, France; 7 Centre Hospitalier Versailles, Service Hospitalo-Universitaire de Psychiatrie de l’Adulte et d’Addictologie, Le Chesnay, France; 8 Université Versailles-Saint-Quentin-en-Yvelines, Versailles, France; Duke University Medical Center: Duke University Hospital, UNITED STATES

## Abstract

**Background:**

Most studies published to date have investigated the impact of the COVID-19 pandemic on suicidal acts using hospital data. Trends from primary care in a country such as France are crucial, as individuals may not consult hospital services after suicide attempts (SAs) but rather see their general practitioner (GP).

**Objectives:**

We aimed to evaluate whether the incidence and characteristics of SAs and completed suicides (CSs) reported to French GPs were different during the COVID-19 pandemic than those of before.

**Methods and findings:**

We conducted a retrospective observational study using data from a nationwide monitoring system, the French Sentinel Network (FSN). All SAs and CSs reported by GPs to the FSN from January 1, 2010, to March 10, 2022 were included. The annual incidence rates (IRs) and the characteristics of SAs and CSs during the pandemic (March 11, 2020, to March 10, 2022) were compared to those of before. In total, 687 SAs and 169 CSs were included. The IRs remained stable for SAs and CSs before and during the pandemic (overlap in confidence intervals). The mean IRs were 52 (95%CI = 44; 57) per 100,000 inhabitants for SAs during the pandemic *versus* 47 [36; 57] during the pre-pandemic period (*p* = 0.49), and 5 (95%CI = 2; 9) for CSs *versus* 11 [6; 16] (*p* = 0.30). During the pandemic, SA were slightly different from those before in terms of age and occupational status (young/students and older/retirees over-represented), history of consultation and expression of suicidal ideas to GP (more frequent), and CS in terms of occupational status (students over-represented) (p<0.05).

**Conclusion:**

The COVID-19 pandemic had no major effect on the overall incidence of SAs and CSs reported to French GPs. However, more suicidal acts were reported among younger and older individuals. Suicidal patients and GPs have adapted by improving the expression of suicidal ideas.

## Introduction

The coronavirus disease (COVID-19) pandemic has altered all aspects of daily life. This led experts to raise alarms about a potential increase in suicidal acts during the pandemic [1]. Suicide attempts (SAs) and completed suicides (CSs) are associated with well-recognized demographic, psychiatric, familial, socio-economic, and societal factors [2]. Their prevention requires interventions at all levels of society, from the community to primary care to specialized care [3].

Most large-scale longitudinal studies have found that SA and CS rates were unchanged or decreased during the early phase of the pandemic [4–16]. An initial decrease in CSs was previously described as a “honeymoon period” [17] or “pulling-together phenomenon” [18]. Increase in suicide rates were then reported in late 2020 and early 2021 in Japan and in suicide attempts among adolescents in the United States [7, 19]. The few studies that have reported data beyond Spring 2021 have shown mixed results [20, 21]. A number of authors reported increases in SA or CS rates among females [7, 19, 20], children, and adolescents [7, 14, 19, 20, 22]. However, most studies were limited to the early stages of the pandemic and provided limited information on living conditions which limits the interpretation of the results [20].

Studies examining the impact of the COVID-19 pandemic on suicidal acts in general practice are scare [13, 14, 23]. General practitioners (GPs) have a central role in suicide prevention [24], which was equally true during the pandemic [25]. Moreover, in a country such as France, GPs play a crucial role in the management of patients after a SA. A study from a representative sample of the French population showed that 39.3% of individuals did not visit a hospital (53.4% of 18–24 year-olds) after a SA [26]. Among them, 37.7% reported visiting a doctor or a psychiatrist/psychologist. Another study showed that French GPs were more likely to be involved in the management of the patient at the time of the SA if the patients were younger [27]. This may have been exacerbated during the pandemic due to hospitals being overwhelmed or the fear of going to the hospital.

In France, an overall 8.5% decrease in the total number of self-harm hospitalizations was reported during the COVID-19 pandemic from September 2020 to August 2020 [16]. Its remains at lower level than expected until August 2021 [20]. On the contrary, the number of calls for intentional drug or other toxic ingestions to the French poison control centers were above what was expected during the COVID period [28]. These studies reported differences according to age and gender, with young females being particularly affected by the persistance of the pandemic. Evidence is currently lacking, but it is likely that the numbers and characteristics of SAs and CSs among primary care patients have changed during the pandemic.

Since 1999, GPs participating in the French Sentinel Network (FSN), a nationwide, near real-time monitoring system, have reported SAs and CSs occurring among their patients. Using data from the FSN, we aimed to compare incidence rates and characteristics of SAs and CSs reported by French GPs during the two years of COVID-19 pandemic to those of the preceding ten years. To account for potential changes during the pandemic, we also compared the SAs and CSs reported during the first year of the pandemic (stringent restrictions) with those of the second year (eased restrictions).

## Materials and methods

Study design and settings A retrospective observational study was performed in France using data collected between March 11, 2010, and March 10, 2022, from the FSN. The first year of the pandemic in France (March 11, 2020 to March 10, 2021) included two long national lockdowns (altogether, three months and ten days), two curfews (altogether, three months), and the prohibition of collective activities and closure of the associated venues (from seven months to one year, depending on the activity); the second year (March 11, 2021, to March 10, 2022) included a short lockdown (eighteen days) followed by the progressive reopening of venues for leisure activities, and access to vaccination (S1 Table). While the lockdowns were nationwide, the level of contamination and deaths varied greatly between administrative regions: during the early part of the pandemic, the Île-de-France and the North-East had the highest reported cumulative rates of hospitalization or death from COVID-19, and the South-Est had a low reported rate.

### French primary care system particularities and data source

France provides universal health insurance for its population under a system that reimburses GPs on the basis of a national fee schedule. This primary healthcare system offers coverage for all residents across the country. GPs do not serve a defined practice population: primary care offers coverage for all residents, and all residents may freely choose their GP. Since 1984, the FSN, a nationwide monitoring system, has collected near real-time epidemiological data from participating French GPs. For the purpose of this study, we used data on SAs and CSs reported by GPs to the FSN.

The FSN comprises approximately 500 voluntary Sentinel GPs (1.2% of the French GP population) who routinely report data on health indicators, including suicidal acts. Sentinel GPs are similar to the overall French GP population in terms of age, and the network covers all regions [29]. Sentinel GPs differ from French GPs in terms of sex and geographical distribution, but estimated incidence rates are corrected for bias due to the absence of geographical representativeness by estimating the weighted incidence using external information about the medical population per region each year [30].

### Data collection, case definition and inclusion criteria

Each week, GPs report the number of suicidal acts among their patients through an online questionnaire. GPs report each suicidal act in their patients whether they were reported by the patient him/herself or by other professional caregivers or their family. Suicidal acts are defined as follows: “self-inflicted injury or self-poisoning with drugs in excess of the generally recognized therapeutic dose, excluding non-suicidal self-injury or self-poisoning [31, 32]”.

For each reported case, GP provide descriptive data. Collected data evolve according to research interests and context [27, 31, 33, 34]. The data included sociodemographic characteristics, history of previous attempts, the method used for the suicidal act, the vital outcome (whether the patient survived or died), and the characteristics of the last consultation in primary care (date, reasons, exploration, and expression of suicidal ideation). From January 2020, data on psychiatric disorders, psychosocial context (job insecurity, financial, sentimental, familial difficulties, social isolation), and potential links between the suicidal acts and the pandemic have also been collected.

All cases reported by Sentinel GPs from March 11, 2010, to March 10, 2022 were included in the analysis, except those for which the vital outcome of the suicidal act was missing (n = 64).

### Variables

The outcomes of this study were the incidence rates for SAs and CSs reported to the French GPs during the COVID-19 pandemic in comparison with the pre-pandemic period. SAs and CSs were defined according to the vital outcome of the suicidal acts reported by the Sentinel GPs. We defined the COVID-19 pandemic period from March 11, 2020, (the World Health Organization declared the global pandemic on that day) to March 10, 2022. The ‘pre-pandemic period’ was defined from March 11, 2010, to March 10, 2020, as the reference period. Age groups were categorized according to predefined limits (≤ 25, 26–65, > 65). Suicide methods were grouped into a single dummy ‘violent method: yes/no’ variable according to previous research (“non-violent methods” included self-poisoning by pharmacological agents ± alcohol, gases, or other toxic substances, and “violent methods” included hanging, firearms, self-cutting, jumping from a height, crashing a car, or jumping or lying in front of a train or a car) [35]. Two geographical variables were created from the zip code of the GP who reported SAs and CSs during the periods of interest. The first was a five-category variable, defined according to the telephone area code (Ile-de-France, Northwest, Northeast, Southeast, Southwest), consistent with the various degrees of pandemic intensity across the French regions as described in the ‘settings’ subsection [36]. The second was a rural/urban dummy variable created from the zip code of the GPs based on data from the French National Statistical Institute (INSEE, France).

### Statistical analysis

We estimated the national incidence rates of SA and CS by multiplying the average number of cases per Sentinel GPs (adjusted for GP participation and geographical distribution) by the total number of GPs in France and then divided by the French population [30]. Calculation of the 95% confidence interval (95%CI) was based on the assumption that the number of reported cases follows Poisson’s distribution. Significance of trends was analyzed by plotting estimated annual incidence rates over years and assessing overlap of confidence intervals. We considered the incidence rates significantly different if no overlap in confidence intervals were present. Average annual incidence rates for before *vs* during the pandemic were compared using the Mann-Whitney test. The characteristics of participating GPs and the SAs/CSs before *vs* during the pandemic were compared using Fischer tests for categorical variables and Student tests for continuous variables. Missing data were not included in percentage calculations. *P*-values < 0.05 were considered statistically significant. Analyses were performed using R software version 3.3.1093.

### Ethical statements

The FSN is approved by the National Data Protection Agency (CNIL, registration number #47139). The protocol was conducted in agreement with the Helsinki Declaration. All GP participants are volunteers to participate. They are informed about studies conducted from their medical charts. Patients are informed that their GP belongs to the FSN. Data reported by GP to the FSN are anonymous. We performed this study on anonymous data without any way to identify patients. For this kind of studies, under French law, consent of the patients is not needed. But patients are well informed that they can refuse the transmission of their data. The ethics committee « Comité de protection des personnes Ile de France V » approved this procedure.

## Results

In total, 1,265 SAs and 348 CSs were reported to the FSN during the study period, including 325 SAs and 75 CSs during the COVID-19 pandemic and 940 SAs and 273 CSs during the preceding period.

Sentinel GPs during the pandemic differed from those of the preceding period in terms of sex (more women during the pandemic), age (younger), and geographical area (Ile-de-France over-represented, Southeast under-represented) (S2 Table). They did not differ in terms of medical practice and or urban/rural status. Sentinel GPs during the first and the second year of the pandemic had similar characteristics (S1 Table).

**Fig 1:**
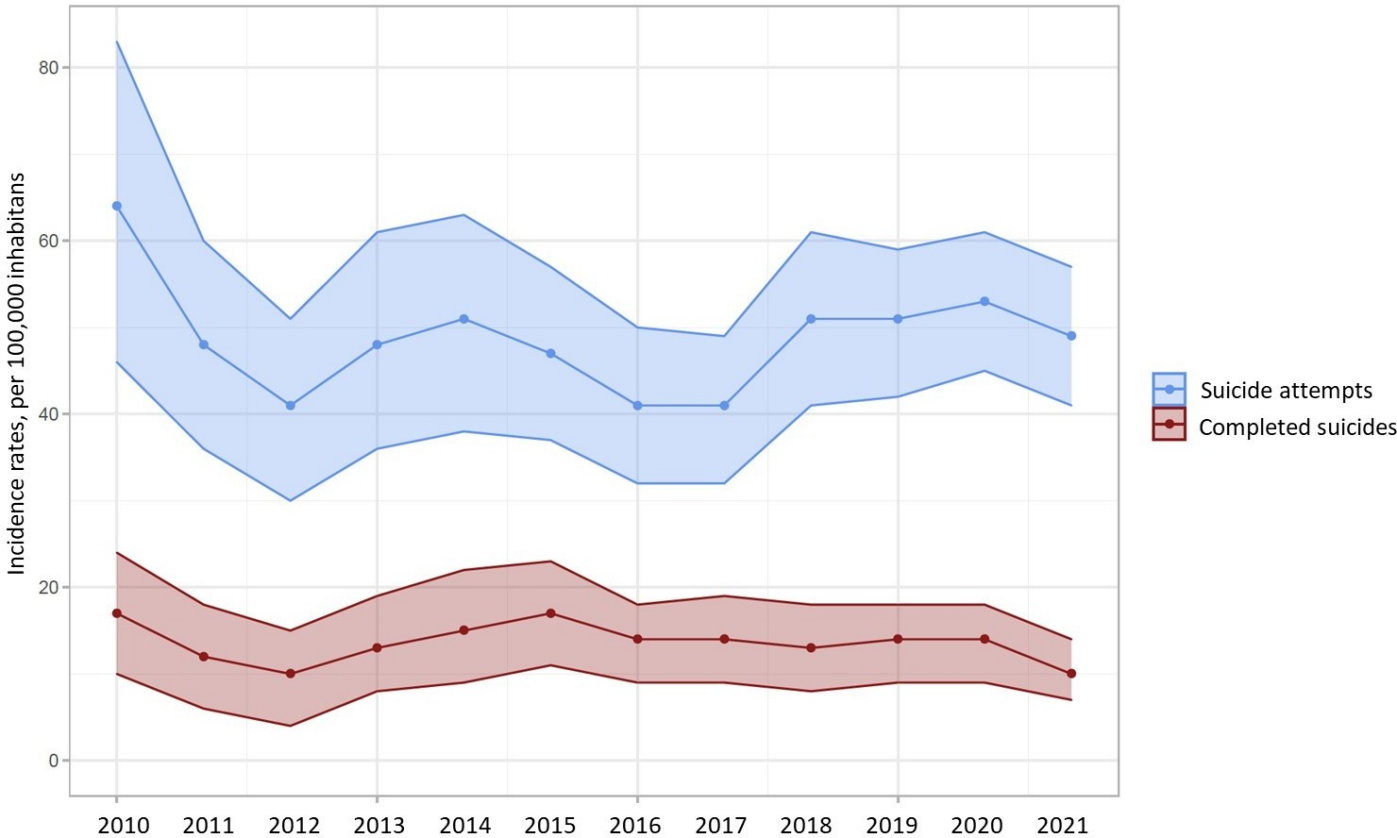


The annual number of SAs and CSs among the French GP patients during the pandemic were estimated to be 31,235 (95%CI = 26,327; 36,146) and 7,303 (95%CI = 4,906; 9,700), respectively. Annual incidence rates for SA and CS between 2010–2021 are showed in Fig 1. Detailed data are provided in S3 Table. Between 2010–2022, the annual incidence rates remained stable for SAs and CSs (no overlap in confidence intervals, Fig 1 and S3 Table). For SA, the mean annual incidence rates were of 52 (95%CI = 44; 57) per 100,000 inhabitants during the pandemic *versus* 47 (95%CI = 36; 57) before the pandemic (*p* = 0.49). For CS, the mean annual incidence rates were of 5 (95%CI = 2; 9) *versus* 11 (95%CI = 6; 16; *p* = 0.30) (Table 1).

**Table 1:** Comparison of the mean annual incidence rates (per 100,000) of attempted and completed suicide reported to the French GPs during the COVID-19 pandemic (March 11, 2020, to March 10, 2022) and before (March 11, 2010 to March 10, 2020), French General Practice Sentinel Network.

	Number of cases reported to Sentinel GP	Mean annual incidence rates per 100,000 inhabitants (95%CI)		*p*-value
		Pre-pandemic (March, 2010-March 2020)	Covid-19 pandemic (March, 2010-March 2020)	
Suicide attempts	1,265	47 (36.57)	52 (44; 57)	0.49
Completed suicides	348	11 (6; 16)	5 (2; 9)	0.30

Among SAs, individuals more highly represented during the pandemic than the preceding period were those in the youngest (≤25) and oldest (over 65) age group (*p* < 0.0001), students and retirees (*p* < 0.0001), those in the Northwest and Southwest of France (*p* = 0.003), those with a history of consultation (*p* = 0.007), and those who spontaneously expressed suicidal ideas (SIs) (*p* < 0.001) (Table 2). GPs more frequently explored SIs among patients who made a SA during the pandemic (*p* < 0.001). Other characteristics (sex, urban/rural status, suicidal method, time since last consultation and reasons) were not statistically different.

**Table 2:** Comparison of suicide attempters and completers reported to the Sentinel GPs during the COVID-19 pandemic (from March 11, 2020, to March 10, 2022) and the preceding period (from March 11, 2010, to March 10, 2020), French General Practice Sentinel Network.

	Suicide attempters			Suicide completers		
	Pre-pandemic N = 940	Pandemic N = 325	*P*-value	Pre-pandemic N = 273	Pandemic N = 75	*P*-value
	n (%)	n (%)		n (%)	n (%)	
Male N (%)	391 (42.1)	129 (40.6)	0.65	199 (73.7)	52 (69.3)	0∙47
Age (years)			<0.0001			0∙37
≤ 25	203 (26.6)	106 (32.6)		17 (6.2)	5 (6.7)	
26–65	643 (68.4)	49 (51.4)		152 (55.7)	48 (64.0)	
> 65	94 (10.0)	52 (16.0)		104 (38.1)	22 (29.3)	
Occupational status			<0.0001			0.03
Workers	309 (45.6)	108 (34.6)		68 (36.2)	23 (32.9)	
Students	100 (14.8)	60 (19.2)		4 (2.1)	3 (4.3)	
Unemployed	114 (16.8)	21 (6.7)		24 (12.8)	9 (12∙9)	
Retirees	93 (13.7)	55 (17∙6)		84 (44.7)	24 (34∙3)	
Other	61 (9.0)	68 (21.9)		8 (4.3)	11 (15.7)	
Geographical area in France			0∙003			0∙17
Ile de France	94 (10.0)	30 (9.2)		22 (8.1)	10 (13.3)	
Northeast	238 (25.3)	80 (24.6)		55 (20.2)	19 (25.3)	
Northwest	202 (21.5)	84 (25.9)		59 (21.6)	20 (26.7)	
Southeast	310 (33.0)	78 (24.0)		91 (33.3)	17 (22.7)	
Southwest	96 (10.2)	53 (16.3)		46 (16.9)	9 (12.0)	
Urban (vs rural)	712 (75.7)	263 (80.9)	0.06	180 (65.9)	54 (72.0)	0∙34
History of previous attempts	356 (41.0)	124 (40.1)	0∙84	64 (28.0)	22 (34.9)	0∙28
Suicidal methods			0∙07			0∙29
Drugs ± alcohol	621 (68.2)	198 (62.3)		34 (13.0)	7 (10.0)	
Hanging	66 (7.2)	38 (12∙0)		117 (44.7)	34 (48.6)	
Firearm	18 (2.0)	6 (1.9)		51 (19.5)	8 (11.4)	
Self-cutting	82 (9.0)	25 (7.9)		4 (1.5)	0 (0)	
Others/multiple	124 (13.6)	51 (16.0)		56 (21.4)	21 (30.0)	
Violent suicidal methods (vs non-violent)	201 (24.0)	83 (27.6)	0∙22	203 (85.3)	46 (80.7)	0∙42
History of consultation	780 (84.4)	293 (90.4)	0.007	183 (68.0)	58 (78.4)	0.22
Time since the last consultation			0.74			
<1 week	138 (18.1)	52 (18.0)		37 (20.4)	9 (15.5)	
1–4 weeks	284 (37.3)	101 (35.0)		62 (34.3)	15 (25.9)	
1> months	340 (44.6)	136 (47.1)		82 (45.3)	34 (58.6)	
Reasons for the last consultation						
Somatic	162 (40.9)	129 (44.0)	0.44	36 (40.9)	32 (55.2)	0.13
Psychological	193 (48.7)	149 (50.9)	0.59	27 (30.7)	17 (29.3)	1
Chronic disease	94 (23.7)	68 (23.2)	0.93	36 (40.9)	19 (32.8)	0.38
Others	33 (8.3)	17 (5.8)	0.24	5 (5.7)	8 (13∙8)	0∙14
Suicidal ideas spontaneously expressed	123 (16.1)	73 (25.4)	<0.001	36 (19.9)	12 (20.7)	0.85
Suicidal ideas explored by the GP	264 (45.8)	170 (59.0)	<0.001	58 (55.2)	26 (44.8)	0.75
Suicidal ideas expressed after GP’s exploration	33 (44.0)	75 (45.2)	0.89	6 (35.3)	12 (52.2)	0∙35

Missing values for sex (SAs: n = 19; CSs: n = 3), occupational status (SAs: n = 276; CSs: n = 90), history of previous attempts (SAs: n = 88; CSs: n = 56), suicidal methods (SAs: n = 36; CSs: n = 16), history of consultation (SAs: n = 17; CSs: n = 5), time since last consultation (SAs: n = 19; CSs: n = 2), reasons for the last consultation (SAs: n = 384; CSs: n = 95), suicidal ideas spontaneously expressed (SAs: n = 198; CSs: n = 2), suicidal ideas explored by the GPs (SAs: n = 384; CSs: n = 46), suicidal ideas expressed after GP’s exploration (SAs: n = 193; CSs: n = 34).

Among CSs, students and others (mainly homemakers and young children) were more highly represented during the pandemic than the preceding period (*p* = 0.03, Table 2). No difference was found for the other characteristics.

GPs identified psychiatric disorders for 69.6% and 71.2% of the SAs and SCs, respectively. The most frequent psychiatric disorder among both SA and CS was depression or mood disorders (77.5% and 73.0%, respectively), followed by anxiety (33.8% and 35.1%, respectively), and substance use disorders (25.8% and 18.9%, respectively). According to GP, 15.9% of SAs and 31.1% of CSs had at a least partial link with the COVID-19 pandemic. Among them, the most frequent COVID-related factor was social isolation for both SA (67.7%) and CS (71.4%).

Among SAs, males were more highly represented during the first year of the pandemic than during the second year (*p* = 0.008), as were those in the Northeast (while less represented in Southeast, *p* = 0.03), those with substance use disorders (*p* = 0.007), and those experiencing major life events in the previous twelve months (*p* = 0.04) (Table 3).

**Table 3:** Comparison of suicide attempters and completers reported to the Sentinel GP during the first year of COVID-19 pandemic (from March 11, 2020, to March 10, 2021) and the second year (from March 11, 2021, to March 10, 2022), French General Practice Sentinel Network.

Variables*	Suicide attempters					Suicide completers				
	1^st^ year N = 185		2^nd^ year N = 140		*P*-value	1^st^ year N = 41		2^nd^ year N = 34		*P*-value
	N	n (%)	N	n (%)		N	n (%)	N	n (%)	
Male N (%)	183	86 (47.0)	135	43 (31.9)	0.008	41	24 (58.5)	34	28 (82.4)	0∙04
Geographical area in France	185		140		0∙03					ns
Ile de France		17 (9.2)		13 (9.3)						
Northeast		55 (29.7)		25 (17.9)						
Northwest		46 (24.9)		38 (27.1)						
Southeast		34 (18.4)		44 (31.4)						
Southwest		33 (17.8)		20 (14.3)						
Substance use disorders (yes)		34 (28.3)		12 (12.5)	0∙007					ns
Life events in the past 12 months	167	91 (54.5)	125	53 (42.4)	0.04					ns

*The following variables were also tested but not statistically significant (p≥0.05): age (≤ 25/26-65/> 65 years), employment status (workers/students/unemployed/retirees/other), urban (*versus* rural), history of previous attempts (yes/no), suicidal methods (drugs±alcohol/hanging/firearm/self-cutting/others or multiple), violent suicidal methods (yes/no), history of consultation (yes/no), time since last consultation (< 1/1-4/> 4 weeks), reasons for the last consultation (somatic/psychological/chronic disease/others), suicidal ideas (SIs) spontaneously expressed (yes/no), SI explored by the GP (yes/no), SIs expressed after GP’s exploration (yes/no), relationship status (couple/single/other), psychiatric disorders (yes/no), depression or mood disorders (yes/no), anxiety (yes/no), personality disorders (yes/no), life problems (yes/no). Details are presented in S1 Table.

ns: not statistically significant.

Suicide completers were less frequently men during the first year of the pandemic than during the second year (*p* = 0.04; Table 3). There was no difference for the other characteristics. Details are presented in S4 Table.

## Discussion

This is the first study to examine how the COVID-19 pandemic influenced SAs and CSs among patients in primary care during the first two years. Using data from a nationwide monitoring system, we found stable SAs and CSs incidence rates during the pandemic relative to the ten preceding years. The two years of the COVID-19 pandemic had only a minor impact on the characteristics of SAs and CSs: they were similar to those shown by prior research before the pandemic, although slight differences were found in terms of age and occupational status. Both patients and GPs exchanged about SIs more frequently during the pandemic compared to before. Suicidal acts were similar during the first and second year of the pandemic, except for differences due to sex (SAs and CSs) and geographical area (SAs).

The COVID-19 pandemic did not increase the incidence of SA and CS reported to the French GP, consistent with results of previous studies conducted in high-income and upper-middle-income [37]. During the initial phase of the pandemic, stable or decrease incidence for CS were also reported in Norway [6], Finland [38], England [39], Ireland [40], Germany [41], Greece [42], Sweden [43] and Austria [44]. Similar results were showed for self-harm presentations to hospitals in the United-Kingdom [45], Spain [46], Portugal [47], France [16], from primary care in the UK [13, 14], and from poison control centers in France [20]. Thus, studies conducted with data from different sources, i.e. hospital, primary care, and poison control centers, suggest a true decrease in the number of suicidal acts during the first part of the pandemic. The absence of an increase in suicidal acts during the pandemic could be explained by better social support, financial help provided by governments, and reduced stress related to school or work. Although we found stable SA and CS rates, it is possible that trends may vary according to age and sex groups. In Japan and France, a decrease of SAs or CSs during the first months of the pandemic followed by an increase in young females has been reported [7, 20]. Similar results were reported from the French poison control center data [28]. Studies conducted by Public Health France in a representative sample of the French adult general population reported high rates of depression, anxiety, sleep problems, and suicidal ideas during the first two years of the pandemic at levels superior to the pre-COVID period. These results highlight the importance of monitoring depressive/anxiety symptoms, and suicidal ideation, in particular in primary care.

In accordance with the results of previous studies, our data suggests that there was higher risk of SA among younger and older individuals, and among students and retirees during the pandemic compared to before [13, 14, 16, 19, 22, 48]. In France, an increase in hospitalization for self-harm was found in adolescent girls, notably between January 2021 and August 2021, while middle-aged adults showed a decrease [20]. An increase in calls to the poison control center from mid-2020 to May 2020 was also observed in young females, and in in older-aged people [28]. Younger individuals and students were particularly affected by the altered school calendar during the pandemic [7, 49] and significant disruptions in their social environment [50, 51]. Older individuals were more concerned by severe COVID-19, and restrictions concerning visits and travel particularly favored social isolation for this group, especially for those living alone [52, 53]. While the unemployment risk factor may have been somewhat mitigated by better financial and social support, whether the pandemic have reduced work-related stress for certain people should be elucidated.

Our data show that classical characteristics (i.e. SAs more likely to be made by females and CSs by males) were erased during the first year of the pandemic. This is in accordance with the results of studies that found an increase in self-harm rates among women in hospital settings at that time [7, 19, 20, 54]. Women may have used more severe lethal methods, leading them to visit the hospital rather than a primary care physician (for SAs), or resulting in a CS. Such a sex-based difference has been attributed to stress, which has been proven to be a specific female suicide factor, as well as emotional problems or peer relationship difficulties [55]. The absence of difference that we found in terms of the methods of SAs or CSs contrasts with those of studies conducted in hospital settings [9, 16]. It is possible that the methods used in SAs seen in general practice may be less affected by severity than those in hospital settings.

The geographical difference we found for SAs between the first and the second year of the pandemic is consistent with the regions where the level of SARS-CoV-2 contamination was higher (first year: Northeast; second year: Southeast) [56]. In light of a previous study that reported a weak and negative correlation between self-harm hospitalizations and COVID-19 hospitalizations across France, our results suggest that patients may have been seen in primary care instead of hospitals in region highly affected by the COVID-19. However, we did not find difference for Ile-de-France, whereas it was one of the regions with the highest reported cumulative rates of hospitalization or death from COVID-19 during the early part of the pandemic. It may be explained by the variation of the French population’s standard of living throughout the country. Indeed, poorest administrative departments are located in the north and on part of the Mediterranean coast, while 43% of those belonging to a very high-income French household, i.e. the wealthiest 1% in the country, resided in Île-de-France, a region immediately surrounding the capital of France, Paris. More studies are needed to explore the impact of the COVID-19 pandemic on suicidal acts according to different socio-economic levels. We also found a geographical difference for SA between the pre-pandemic period and the pandemic. However, our sample of participating GPs were not comparable before *vs* during the pandemic, leading to difficult interpretation.

Our data on the history of consultations and SI expression and exploration are consistent with the crucial role played by GPs in suicide prevention among their patients during the pandemic [25, 57] They are also consistent with data showing that prescribing and consultation patterns in primary care following self-harm in the UK were broadly similar to pre-pandemic levels [23]. It is noteworthy that during the two years of the pandemic, the most frequent psychological factors were life problems and psychiatric disorders but not COVID-19-related stressors [58].

The clinical characteristics of the suicidal acts (psychiatric disorders, stressors) during the COVID pandemic were similar to the already known distribution before the pandemic, whereas the COVID-related stressors were not very frequent in our sample [58]. We found that a history of mental health disorders (particularly depression) was the most frequent factor, in accordance with the results of other studies conducted during the pandemic [59] and before. Psychiatric disorders are the highest risk factor for SAs/CSs, especially depression [58]. We found that the COVID-19 pandemic were identified by the GP as influencing self-harm in only 15.9% of suicide attempters and 31.1% of suicide completers, which is less than that reported in a previous study conducted in England among adults (46.9%) [60] or in the United-States among adolescents (47.2%) [11]. However, the first study was conducted during the first lockdown, when the closure of services and isolation were at their maximum level, and the second was conducted on adolescents, who were particularly concerned by social restrictions. Concerning COVID-19, loneliness was by far the most frequent reason give, in accordance with previous studies [61].

The strengths of this study lie on its wide range of information on primary care for both SAs and CSs (often coming from separate data) and in the use of longitudinal, real-time data collected from a recognized nationwide surveillance system. However, our study had several limitations. First, SAs and CSs may have been underreported by the GPs. Indeed, GPs have reported to the FSN suicidal act in their patients whether they were reported by the patient him/herself or by other professional caregivers or their family. We assume that GPs may not be aware of certain suicidal acts (for patients less involved in general practice). Moreover, GPs might forget to report all cases. We nevertheless found similar SAs:CSs ratios during the different periods, and to those in other general practice networks [62–64]. Second, our sample may not be representative of the GPs in metropolitan France [29]. However, this may have had only a limited impact on our results, as we were more interested in comparing periods, than in making precise estimations. Third, Sentinel GPs during the pandemic differed from those of the preceding years in terms of age, sex, and geographical area. The impact of such differences on reported cases is unknown. Estimated incidence rates are corrected for geographical sampling bias. However, we recognize that the geographical distribution of cases during the pandemic *versus* the preceding period should be interpreted with caution. Fourth, suicidal acts are rare events in a GP’s practice population, and small sample sizes may have led to low statistical power for the detection of differences between periods. This also made not possible the study of trends according to smaller periods or/and to age and sex. Moreover, we recognized that the comparison of mean annual IRs has very limited value. However, given the flat curve of trends for SA/CS incidence rates, we believed that it was not necessary to perform more complicated statistical models to show that the incidence rates were stable during the pandemic compare to before. Again, our small sample size would have limited the use of such models. Fifth, information may have been biased due to the GP not being aware of patient’s history or recall bias. Sixth, we could not examine the impact of the pandemic on psychosocial factors, on psychiatric diagnoses (as this information was not collected before 2020), and on the management of SI expression or of SAs/CSs (as this information has not been collected since 2017). Finally, we did not collect information on COVID-19 status.

In conclusion, there is no major impact of the COVID-19 pandemic on the overall incidence rates of SA and CS reported to the French GPs during the first two years. However, more suicidal acts were reported among younger and older individuals. More studies including data of late 2021 and 2022 are needed to confirm these results.

## Supporting information

S1 Table Measures of restrictions against COVID-19 in France, March 2020-March 2022. (PDF)

S2 Table Comparison of GP in the French General Practice Sentinel Network in the COVID-19 pandemic (from March 11, 2020, to March 10, 2022) and in the preceding period (from March 11, 2010, to March 10, 2020), and in the first year of the pandemic (from March 11, 2020, to March 10, 2021) and the second year (from March 11, 2021, to March 10, 2022). (PDF)

S3 Table Annual incidence rates (per 100,000) of attempted and completed suicide reported to the French GPs between 2010 and 2021. (DOCX)

S4 Table Characteristics of suicide completers and suicide attempters during the first year of COVID-19 pandemic (from March 11, 2020, to March 10, 2021) and the second year (from March 11, 2021, to March 10, 2022) in the French General Practice Sentinel Network. (DOCX)
