# Supplementary material for: Trends and characteristics of attempted and completed suicides reported to general practitioners before vs during the COVID-19 pandemic in France: Data from a nationwide monitoring system, 2010–2022
Source: PLoS One. 2022 Dec 15;17(12):e0278266. doi: 10.1371/journal.pone.0278266 (PMC9754243; doi:10.1371/journal.pone.0278266)
Supplement: S1 Table — (PDF) [file pone.0278266.s001.pdf]

Supplementary Materials

Contents:

Table S1). Measures of restrictions against COVID-19 in France, March 2020-March 2022. 2

Table S1). Measures of restrictions against COVID-19 in France, March 2020-March 2022.

Restrictions	First year (March 2020-March 2021)												Second year (March 2021-March 2022)												
	Mar	Apr	May	Jun	Jul	Aug	Sep	Oct	Nov	Dec	Jan	Fev	Mar	Apr	May	Jun	Jul	Aug	Sep	Oct	Nov	Dec	Jan	Fev	Mar
Lockdowns																									
Curfews																									
Closure of school and university, or distance leaning																									
Distance learning for universities																									
Closure of restaurants, bars, museums, cinema																									
Closure of disco																									
Travel ban or limitation																									
Access to COVID-19 vaccination																									
