# Supplementary material for: Trends and characteristics of attempted and completed suicides reported to general practitioners before vs during the COVID-19 pandemic in France: Data from a nationwide monitoring system, 2010–2022
Source: PLoS One. 2022 Dec 15;17(12):e0278266. doi: 10.1371/journal.pone.0278266 (PMC9754243; doi:10.1371/journal.pone.0278266)
Supplement: S2 Table — (PDF) [file pone.0278266.s002.pdf]

Supplementary Materials

Contents:

Table S2). Comparison of GP in the French General Practice Sentinel Network in the COVID-19 pandemic (from March 11, 2020, to March 10, 2022) and in the preceding period (from March 11, 2010, to March 10, 2020), and in the first year of the pandemic (from March 11, 2020, to March 10 ,2021) and the second year (from March 11, 2021, to March 10 ,2022). 2

Table S2). Comparison of GP in the French General Practice Sentinel Network in the COVID-19 pandemic (from March 11, 2020, to March 10, 2022) and in the preceding period (from March 11, 2010, to March 10, 2020), and in the first year of the pandemic (from March 11, 2020, to March 10 ,2021) and the second year (from March 11, 2021, to March 10 ,2022).

Variables	Pre-pandemic N=1,040		Pandemic N=1,291		*P*-value	First year N=702		Second year N=589		*P*-value
	N	n (%)	N	n (%)		N	n (%)	N	n (%)	
**Male** N (%)	1,040	697 (67.0)	1,291	724 (56.1)	**<0.0001**	702	403 (57.4)	589	52 (54.5)	0.31
**Annual mean age (years), mean (±sd)**		51.5 (±10.3)		48.6 (±11.7)	**0.02**		48.5 (±11.8)		48.6 (±11.7)	
**Medical practice**	802		1,025		0.29	559		466		0.73
Solo practitioner		261 (32.5)		309 (30.1)			166 (29.7)		143 (70.3)	
Group practitioner		541 (67.5)		716 (69.9)			393 (30.7)		323 (69.3)	
**Geographical area in France**	1,040		1,291		**0.0001**	702		589		0.95
Ile de France		123 (11.8)		216 (16.7)			116 (16.5)		100 (17.0)	
Northeast		197 (18.9)		252 (19.5)			134 (19.1)		118 (20.0)	
Northwest		220 (21.2)		282 (21.8)			157 (22.4)		125 (21.2)	
Southeast		365 (35.1)		349 (27.0)			187 (26.6)		162 (27.5)	
Southwest		135 (13.0)		192 (14.9)			108 (15.4)		84 (14.3)	
**Urban (vs rural)**	1,039	814 (78.3)	1,289	995 (77.2)	0.52	701	544 (77.6)	588	451 (76.7)	0.74
