# Supplementary material for: Trends and characteristics of attempted and completed suicides reported to general practitioners before vs during the COVID-19 pandemic in France: Data from a nationwide monitoring system, 2010–2022
Source: PLoS One. 2022 Dec 15;17(12):e0278266. doi: 10.1371/journal.pone.0278266 (PMC9754243; doi:10.1371/journal.pone.0278266)
Supplement: S3 Table — (DOCX) [file pone.0278266.s003.docx]

**Supplementary Materials**

**Contents:**

[Table S3). Annual incidence rates (per 100,000) of attempted and completed suicide reported to the French GPs between 2010 and 2021 2](#_Toc114779422)

# Table S3). Annual incidence rates (per 100,000) of attempted and completed suicide reported to the French GPs between 2010 and 2021.

| **Year** | **Suicide attempts** | | | **Completed suicide** | | |
| --- | --- | --- | --- | --- | --- | --- |
|  | **N** | **Incidence rates per 100,000 inhabitants (95%CI)** | | **N** | **Incidence rates per 100,000 inhabitants (95%CI)** | |
| 2010 | 107 | 64 | (46; 83) | 31 | 17 | (10; 24) |
| 2011 | 90 | 48 | (36; 60) | 24 | 12 | (6; 18) |
| 2012 | 86 | 41 | (30; 51) | 20 | 10 | (4; 15) |
| 2013 | 93 | 48 | (36; 61) | 29 | 13 | (8; 19) |
| 2014 | 89 | 51 | (38; 63) | 28 | 15 | (9;22) |
| 2015 | 117 | 47 | (37; 57) | 41 | 17 | (11;23) |
| 2016 | 109 | 41 | (32; 50) | 39 | 14 | (9; 18) |
| 2017 | 107 | 41 | (32; 49) | 36 | 14 | (9;19) |
| 2018 | 125 | 51 | (41; 61) | 30 | 13 | (8; 18) |
| 2019 | 149 | 51 | (42; 59) | 41 | 14 | (9;18) |
| 2020 | 199 | 53 | (45; 61) | 48 | 14 | (9; 18) |
| 2021 | 166 | 49 | (41; 57) | 37 | 10 | (7; 14) |
