# Supplementary material for: Trends and characteristics of attempted and completed suicides reported to general practitioners before vs during the COVID-19 pandemic in France: Data from a nationwide monitoring system, 2010–2022
Source: PLoS One. 2022 Dec 15;17(12):e0278266. doi: 10.1371/journal.pone.0278266 (PMC9754243; doi:10.1371/journal.pone.0278266)
Supplement: S4 Table — (DOCX) [file pone.0278266.s004.docx]

**Supplementary Materials**

**Contents:**

[Table S4) Characteristics of suicide completers and suicide attempters during the first year of COVID-19 pandemic (from March 11, 2020, to March 10 ,2021) and the second year (from March 11, 2021, to March 10 ,2022) in the French General Practice Sentinel Network. 2](#_Toc114784002)

# Table S4) Characteristics of suicide completers and suicide attempters during the first year of COVID-19 pandemic (from March 11, 2020, to March 10 ,2021) and the second year (from March 11, 2021, to March 10 ,2022) in the French General Practice Sentinel Network.

| **Variables** |  | **Suicide attempters** | | | | | | | |  | **Suicide completers** | | | | | | |
| --- | --- | --- | --- | --- | --- | --- | --- | --- | --- | --- | --- | --- | --- | --- | --- | --- | --- |
|  |  | **1^st^ year N = 185** | | | | **2^nd^ year N = 140** | | | ***P*-value** |  | **1^st^ year N = 41** | | | **2^nd^ year N = 34** | | | ***P*-value** |
|  |  | **N** | | **n** | **(%)** | **N** | **n** | **(%)** |  |  | **N** | **n** | **(%)** | **N** | **n** | **(%)** |  |
| **Male** N (%) |  | 183 | 86 | | (47.0) | 135 | 43 | (31.9) | **0.008** |  | 41 | 24 | (58.5) | 34 | 28 | (82.4) | **0∙04** |
| **Age (years)** |  | 185 |  | |  | 140 |  |  | 0∙05 |  | 41 |  |  | 34 |  |  | 0**∙**38 |
| ≤ 25 |  |  | 51 | | (27.6) |  | 55 | (39.3) |  |  |  | 4 | (9.8) |  | 1 | (2.9) |  |
| 26-65 |  |  | 105 | | (56.8) |  | 62 | (44.3) |  |  |  | 27 | (65.9) |  | 21 | (61.8) |  |
| > 65 |  |  | 29 | | (15.7) |  | 23 | (16.4) |  |  |  | 10 | (24.4) |  | 12 | (35.3) |  |
| **Employment status** |  | 176 |  | |  | 136 |  |  | 0.38 |  | 39 |  |  | 31 |  |  | 0.58 |
| Workers |  |  | 65 | | (36.9) |  | 43 | (31.6) |  |  |  | 14 | (35.9) |  | 9 | (29.0) |  |
| Students |  |  | 27 | | (15.3) |  | 33 | (24.3) |  |  |  | 2 | (5.1) |  | 1 | (3.2) |  |
| Unemployed |  |  | 13 | | (7.4) |  | 8 | (5.9) |  |  |  | 6 | (15.4) |  | 3 | (9.7) |  |
| Retirees |  |  | 31 | | (17.6) |  | 24 | (17∙7) |  |  |  | 10 | (25.6) |  | 14 | (45.2) |  |
| Other |  |  | 40 | | (22.7) |  | 28 | (20.6) |  |  |  | 7 | (18.0) |  | 4 | (12.9) |  |
| **Geographical area in France** |  | 185 |  | |  | 140 |  |  | **0∙03** |  | 41 |  |  | 34 |  |  | 0**∙**35 |
| Ile de France |  |  | 17 | | (9.2) |  | 13 | (9.3) |  |  |  | 5 | (12.2) |  | 5 | (14.7) |  |
| Northeast |  |  | 55 | | (29.7) |  | 25 | (17.9) |  |  |  | 12 | (29.3) |  | 7 | (20.6) |  |
| Northwest |  |  | 46 | | (24.9) |  | 38 | (27.1) |  |  |  | 8 | (19.5) |  | 12 | (35.3) |  |
| Southeast |  |  | 34 | | (18.4) |  | 44 | (31.4) |  |  |  | 12 | (29.3) |  | 5 | (14.7) |  |
| Southwest |  |  | 33 | | (17.8) |  | 20 | (14.3) |  |  |  | 4 | (9.8) |  | 5 | (14.7) |  |
| **Urban (vs rural)** |  | 185 | 147 | | (79.5) | 140 | 116 | (82.9) | 0.48 |  | 41 | 32 | (78.1) | 34 | 22 | (64.7) | 0**∙**30 |
| **History of previous attempts** |  | 175 | 70 | | (40.0) | 134 | 54 | (40.3) | 1 |  | 32 | 10 | (31.3) | 31 | 12 | 38.7 | 0.60 |
| **Suicidal methods** |  | 182 |  | |  | 136 |  |  | 0**∙**13 |  | 41 |  |  | 34 |  |  | 0**∙**31 |
| Drugs ± alcohol |  |  | 99 | | (54.4) |  | 83 | (61.0) |  |  |  | 6 | (15.8) |  | 1 | (3.1) |  |
| Hanging |  |  | 26 | | (14.3) |  | 11 | (8.1) |  |  |  | 16 | (42.1) |  | 18 | (56.3) |  |
| Firearm |  |  | 2 | | (1.1) |  | 4 | (2.9) |  |  |  | 5 | (13.2) |  | 3 | (9.4) |  |
| Self-cutting |  |  | 18 | | (9.9) |  | 7 | (5.2) |  |  |  | 0 | (0) |  | 0 | (0) |  |
| Others/multiple |  |  | 37 | | (20.3) |  | 31 | (22.8) |  |  |  | 11 | (29.0) |  | 10 | (31.2) |  |
| **Violent suicidal methods (vs non-violent)** |  | 160 | 50 | | (31.3) | 116 | 26 | (22.4) | 0.13 |  | 32 | 24 | (75.0) | 25 | 22 | (88.0) | 0.32 |
| **History of consultation** |  | 184 | 164 | | (89.1) | 140 | 129 | (92.1) | 0.45 |  | 41 | 32 | (78.1) | 33 | 26 | (78.8) | 1 |
| **Time since the last consultation** |  | 162 |  | |  | 127 |  |  | 0.99 |  | 32 |  |  | 26 |  |  | 0∙66 |
| <1 week |  |  | 29 | | (17.9) |  | 23 | (18.1) |  |  |  | 6 | (18.8) |  | 3 | (11.5) |  |
| 1-4 weeks |  |  | 56 | | (34.6) |  | 45 | (35.4) |  |  |  | 7 | (21.9) |  | 8 | (20.8) |  |
| 1> months |  |  | 77 | | (47.5) |  | 59 | (46.5) |  |  |  | 19 | (59.4) |  | 15 | (57.7) |  |
| **Reasons for the last consultation** |  | 164 |  | |  | 129 |  |  |  |  |  |  |  |  |  |  |  |
| Somatic |  |  | 71 | | (43.3) |  | 58 | (45.0) | 0.81 |  | 32 | 21 | (65.6) | 26 | 11 | (42.3) | 0.11 |
| Psychological |  |  | 86 | | (52.4) |  | 63 | (48.8) | 0.56 |  | 32 | 11 | (34.4) | 26 | 6 | (23.1) | 0.40 |
| Chronic disease |  |  | 36 | | (22.0) |  | 32 | (24.8) | 0.58 |  | 32 | 10 | (31.3) | 26 | 9 | (34.6) | 1 |
| Others |  |  | 10 | | (6.1) |  | 7 | (5.4) | 1 |  | 32 | 3 | (9.4) | 26 | 5 | (18.2) | 0.45 |
| **Suicidal ideas spontaneously expressed** |  | 161 | 38 | | (23.6) | 127 | 35 | (27.6) | 0.50 |  | 35 | 8 | (22.9) | 13 | 3 | (23.1) | 1 |
| **Suicidal ideas explored by the GP** |  | 161 | 94 | | (58.4) | 127 | 76 | (59.8) | 0.81 |  | 35 | 19 | (54.3) | 13 | 5 | (38.5) | 0.52 |
| **Suicidal ideas expressed after GP’s exploration** |  | 92 | 41 | | (44.6) | 74 | 34 | (46.0) | 0.88 |  | 17 | 9 | (52.9) | 4 | 2 | (50.0) | 1 |

**Table S3) (continued)**

|  |  | **Suicide attempters** | | | | | | |  | **Suicide completers** | | | | | | |
| --- | --- | --- | --- | --- | --- | --- | --- | --- | --- | --- | --- | --- | --- | --- | --- | --- |
|  |  | **1^st^ year N = 185** | | | **2^nd^ year N = 140** | | | ***P*-value** |  | **1^st^ year N = 41** | | | **2^nd^ year N = 34** | | | ***P*-value** |
|  |  | **N** | **n** | **(%)** | **N** | **n** | **(%)** |  |  | **N** | **n** | **(%)** | **N** | **n** | **(%)** |  |
| **Relationship status** |  | **177** |  |  | **129** |  |  | **0.38** |  | **38** |  |  | **33** |  |  | **0.74** |
| Couple |  |  | 84 | (47.5) |  | 53 | (41.1) |  |  |  | 18 | (47.4) |  | 14 | (42.4) |  |
| Single |  |  | 62 | (35.0) |  | 46 | (35.7) |  |  |  | 14 | (36.8) |  | 11 | (33.3) |  |
| Other (divorce proceeding or recent widowhood) |  |  | 31 | (17.5) |  | 30 | (23.3) |  |  |  | 6 | (15.8) |  | 8 | (24.2) |  |
| **Psychiatric disorders (yes)** |  | **177** | **120** | **(67.8)** | **138** | **96** | **(69.6)** | **0.81** |  | **39** | **27** | **(69.2)** | **31** | **17** | **(54.8)** | **0.32** |
| **Depression or mood disorders (yes)** |  | **120** | **89** | **(74.2)** | **96** | **81** | **(84.4)** | **0.09** |  | **27** | **18** | **(66.7)** | **17** | **15** | **(88.2)** | **0.16** |
| **Anxiety (yes)** |  |  | **40** | **(33.3)** |  | **33** | **(34.4)** | **0.86** |  |  | **10** | **(37.0)** |  | **4** | **(23.5)** | **0.51** |
| **Substance use disorders (yes)** |  |  | **34** | **(28.3)** |  | **12** | **(12.5)** | **0∙007** |  |  | **6** | **(22.2)** |  | **2** | **(11.8)** | **0.45** |
| **Personality disorders (yes)** |  |  | **20** | **(16.7)** |  | **16** | **(16.7)** | **1** |  |  | **5** | **(18.5)** |  | **2** | **(11.8)** | **0.69** |
| **Life problems (yes)** |  | **172** | **163** | **(94.8)** | **123** | **117** | **(95.1)** | **1** |  | **27** | **22** | **(81.5)** | **24** | **23** | **(95.8)** | **0.20** |
| *Within* family and/or emotional life |  | *174* | *130* | *(74.7)* | *128* | *90* | *(70.3)* |  |  | *29* | *17* | *(58.6)* | *28* | *18* | *(64.3)* |  |
| *Social isolation* |  | *177* | *52* | *(29.4)* | *130* | *32* | *(24.6)* |  |  | *33* | *8* | *(24.2)* | *31* | *6* | *(19.4)* |  |
| *Within workplace/school* |  | *125* | *53* | *(42.4)* | *87* | *38* | *(43.7)* |  |  | *22* | *7* | *(25.0)* | *15* | *1* | *(4.8)* |  |
| *Financial problems* |  | *152* | *33* | *(21.7)* | *119* | *25* | *(21.0)* |  |  | *30* | *9* | *(30.0)* | *24* | *4* | *(16.7)* |  |
| **Life events in the past 12 months (yes)** |  | **167** | **91** | **(54.5)** | **125** | **53** | **(42.4)** | **0.04** |  | **31** | **20** | **(64.5)** | **31** | **21** | **(67.7)** | **1** |
| *Divorce or separation* |  | *91* | *39* | *(42.9)* | *53* | *21* | *(39.6)* |  |  | *20* | *5* | *(25.0)* | *21* | *5* | *(23.8)* |  |
| *Loss of a parent or of a loved-one* |  |  | *14* | *(15.4)* |  | *10* | *(18.9)* |  |  |  | *3* | *(15.0)* |  | *4* | *(19.1)* |  |
| *Intimidation, harassment or humiliation* |  |  | *9* | *(9.9)* |  | *7* | *(13.2)* |  |  |  | *2* | *(10.0)* |  | *0* | *(0)* |  |
| *Loss of job* |  |  | *5* | *(5.5)* |  | *7* | *(13.2)* |  |  |  | *2* | *(10.0)* |  | *0* | *(0)* |  |
| *Physical aggression or sexual harassment* |  |  | *8* | *(8.8)* |  | *6* | *(11.3)* |  |  |  | *1* | *(5.0)* |  | *0* | *(0)* |  |
| *Other* |  |  | *42* | *(46.2)* |  | *16* | *(30.2)* |  |  |  | *11* | *(55.0)* |  | *13* | *(61.9)* |  |
| **Link with COVID-19 pandemic (yes)** |  | **157** | **25** | **(15.9)** | **129** | **13** | **(10.1)** | **0.16** |  | **31** | **11** | **(35.5)** | **29** | **5** | **(17.2)** | **0.15** |
| *Social isolation* |  | *25* | *17* | *(68.0)* | *13* | *7* | *(53.9)* |  |  | *11* | *7* | *(63.6)* | *5* | *4* | *(80.0)* |  |
| *Family stress* |  |  | *10* | *(40.0)* |  | *2* | *(15.4)* |  |  |  | *4* | *(36.4)* |  | *1* | *(20.0)* |  |
| *Fear of the COVID-19* |  |  | *2* | *(8.0)* |  | *0* | *(0)* |  |  |  | *3* | *(27.3)* |  | *1* | *(20.0)* |  |
| *Material and financial consequences* |  |  | *3* | *(12.0)* |  | *1* | *(7.7)* |  |  |  | *1* | *(9.1)* |  | *0* | *(0)* |  |
| *COVID-19 illness or Loss of a loved-one to COVID-19* |  |  | *3* | *(12.0)* |  | *1* | *(7.7)* |  |  |  | *0* | *(0)* |  | *0* | *(0)* |  |
| *Other* |  |  | *5* | *(20.0)* |  | *3* | *(23.1)* |  |  |  | *2* | *(18.2)* |  | *0* | *(0)* |  |
