# Supplementary material for: Trends and characteristics of attempted and completed suicides reported to general practitioners before vs during the COVID-19 pandemic in France: Data from a nationwide monitoring system, 2010–2022
Source: PLoS One. 2022 Dec 15;17(12):e0278266. doi: 10.1371/journal.pone.0278266 (PMC9754243; doi:10.1371/journal.pone.0278266)
Supplement: Peer review history

# Peer review: Decision Letter 0

**DOI:** 10.1371/journal.pone.0278266.r001

**Authors:** Rikinkumar S. Patel

**Transfer Alert**

This paper was transferred from another journal. As a result, its full editorial history (including decision letters, peer reviews and author responses) may not be present.

8 Aug 2022

PONE-D-22-18701Trends and characteristics of suicidal behaviors in general practice during two years of COVID-19 pandemic in comparison with ten years before: data from the French Sentinel networkPLOS ONE

Dear Dr. Pouquet,

Thank you for submitting your manuscript to PLOS ONE. After careful consideration, we feel that it has merit but does not fully meet PLOS ONE’s publication criteria as it currently stands. Therefore, we invite you to submit a revised version of the manuscript that addresses the points raised during the review process.

Please submit your revised manuscript by Sep 22 2022 11:59PM. If you will need more time than this to complete your revisions, please reply to this message or contact the journal office at plosone@plos.org. When you're ready to submit your revision, log on to https://www.editorialmanager.com/pone/ and select the 'Submissions Needing Revision' folder to locate your manuscript file.

Please include the following items when submitting your revised manuscript:

- A rebuttal letter that responds to each point raised by the academic editor and reviewer(s). You should upload this letter as a separate file labeled 'Response to Reviewers'.
- A marked-up copy of your manuscript that highlights changes made to the original version. You should upload this as a separate file labeled 'Revised Manuscript with Track Changes'.
- An unmarked version of your revised paper without tracked changes. You should upload this as a separate file labeled 'Manuscript'.

If you would like to make changes to your financial disclosure, please include your updated statement in your cover letter. Guidelines for resubmitting your figure files are available below the reviewer comments at the end of this letter.

If applicable, we recommend that you deposit your laboratory protocols in protocols.io to enhance the reproducibility of your results. Protocols.io assigns your protocol its own identifier (DOI) so that it can be cited independently in the future. For instructions see: https://journals.plos.org/plosone/s/submission-guidelines#loc-laboratory-protocols. Additionally, PLOS ONE offers an option for publishing peer-reviewed Lab Protocol articles, which describe protocols hosted on protocols.io. Read more information on sharing protocols at https://plos.org/protocols?utm_medium=editorial-email&utm_source=authorletters&utm_campaign=protocols.

We look forward to receiving your revised manuscript.

Kind regards,

Rikinkumar S. Patel, M.D., M.P.H.

Academic Editor

PLOS ONE

Journal Requirements:

When submitting your revision, we need you to address these additional requirements.

1. Please ensure that your manuscript meets PLOS ONE's style requirements, including those for file naming. The PLOS ONE style templates can be found at 

https://journals.plos.org/plosone/s/file?id=wjVg/PLOSOne_formatting_sample_main_body.pdf and 

https://journals.plos.org/plosone/s/file?id=ba62/PLOSOne_formatting_sample_title_authors_affiliations.pdf.

2. Please provide additional details regarding participant consent. In the ethics statement in the Methods and online submission information, please ensure that you have specified (1) whether consent was informed and (2) what type you obtained (for instance, written or verbal, and if verbal, how it was documented and witnessed). If your study included minors, state whether you obtained consent from parents or guardians. If the need for consent was waived by the ethics committee, please include this information.

If you are reporting a retrospective study of medical records or archived samples, please ensure that you have discussed whether all data were fully anonymized before you accessed them and/or whether the IRB or ethics committee waived the requirement for informed consent. If patients provided informed written consent to have data from their medical records used in research, please include this information.

3. In your Data Availability statement, you have not specified where the minimal data set underlying the results described in your manuscript can be found. PLOS defines a study's minimal data set as the underlying data used to reach the conclusions drawn in the manuscript and any additional data required to replicate the reported study findings in their entirety. All PLOS journals require that the minimal data set be made fully available. For more information about our data policy, please see http://journals.plos.org/plosone/s/data-availability.

"Upon re-submitting your revised manuscript, please upload your study’s minimal underlying data set as either Supporting Information files or to a stable, public repository and include the relevant URLs, DOIs, or accession numbers within your revised cover letter. For a list of acceptable repositories, please see http://journals.plos.org/plosone/s/data-availability#loc-recommended-repositories. Any potentially identifying patient information must be fully anonymized.

Important: If there are ethical or legal restrictions to sharing your data publicly, please explain these restrictions in detail. Please see our guidelines for more information on what we consider unacceptable restrictions to publicly sharing data: http://journals.plos.org/plosone/s/data-availability#loc-unacceptable-data-access-restrictions. Note that it is not acceptable for the authors to be the sole named individuals responsible for ensuring data access.

We will update your Data Availability statement to reflect the information you provide in your cover letter.

Additional Editor Comments:

Additional recommendations/major revision as below:

ABSTRACT

1. add objectives as a separate subsection

2. Line 43 limitation is not needed here

3. What were the P-values for the differences in IR? Pls specify

INTRODUCTION

1. Can you elaborate more on suicidal behaviors and related consequences in France? As in the US, those are crisis situations where patients go straight to the hospital and are not managed outpatient basis. Authors have included SA/CS which is a major crisis and learning the trend in GPs does not define the overall incidence/trend of suicidality during pandemic vs. other ten years' data. After reading the methods it seems this is a follow-up with patients who had SA/CS. If yes, then pls provide a brief on study design in goals as well as the title needs to be changed.

METHODS

1. I will like the authors to clarify that this is the patient-reported history of CS/SA in goals as well as title

2. Subsection and more defined inclusion and exclusion criteria, variables, and outcomes need to be added. Also, summarize current content in the statistical analysis subsection and the last para under ethical approval.

RESULTS

1. Table 1 pls add p-values to support the difference between both IRs. Recommend creating a table with columns between pre-pandemic and pandemic. Currently, you kept them as rows. Recommend to create a line/bar graph trend of IR from 2010 to 2022 and a linear-by-linear association test to evaluate the p-values across the trend. The flaw in analysis to compare IR between pre and during pandemic needs to be addressed

2. Good work on table 2. Why there are two N's. I assume it shows total data collected for that variable and if you reporting missing data then pls only include "n" in the table and calculate missing data from "N" and report it as footer line.

3. Table 2 and 3 can be merged

DISCUSSION

1. I would like to see data results from either France/ European studies related to similar goals and compare the findings, and if there is inpatient study on IR and related factors on suicide then pls compare the findings.

2. Conclusion needs to be revised after re-analysis as recommended. A very strong statement is made and needs to be rectified.

[Note: HTML markup is below. Please do not edit.]

Reviewers' comments:

Reviewer's Responses to Questions

**Comments to the Author**

1. Is the manuscript technically sound, and do the data support the conclusions?

The manuscript must describe a technically sound piece of scientific research with data that supports the conclusions. Experiments must have been conducted rigorously, with appropriate controls, replication, and sample sizes. The conclusions must be drawn appropriately based on the data presented. 

Reviewer #1: Yes

Reviewer #2: Yes

Reviewer #3: Yes

Reviewer #4: Yes

**********

2. Has the statistical analysis been performed appropriately and rigorously? 

Reviewer #1: Yes

Reviewer #2: Yes

Reviewer #3: Yes

Reviewer #4: Yes

**********

3. Have the authors made all data underlying the findings in their manuscript fully available?

The PLOS Data policy requires authors to make all data underlying the findings described in their manuscript fully available without restriction, with rare exception (please refer to the Data Availability Statement in the manuscript PDF file). The data should be provided as part of the manuscript or its supporting information, or deposited to a public repository. For example, in addition to summary statistics, the data points behind means, medians and variance measures should be available. If there are restrictions on publicly sharing data—e.g. participant privacy or use of data from a third party—those must be specified.

Reviewer #1: Yes

Reviewer #2: Yes

Reviewer #3: Yes

Reviewer #4: No

**********

4. Is the manuscript presented in an intelligible fashion and written in standard English?

PLOS ONE does not copyedit accepted manuscripts, so the language in submitted articles must be clear, correct, and unambiguous. Any typographical or grammatical errors should be corrected at revision, so please note any specific errors here.

Reviewer #1: Yes

Reviewer #2: No

Reviewer #3: Yes

Reviewer #4: Yes

**********

5. Review Comments to the Author

Please use the space provided to explain your answers to the questions above. You may also include additional comments for the author, including concerns about dual publication, research ethics, or publication ethics. (Please upload your review as an attachment if it exceeds 20,000 characters)

Reviewer #1: I believe the research question, and aim of the study has been clearly outlined. The methods in the study design are appropriate to answer the aim. The variables are clearly defined. The results are stated clearly, the date presented in a clear and appropriate way. The conclusion answers the aims of the study. the references seem relevant. I am not aware of any key studies were missed that has been referenced

Reviewer #2: Overall, this is a well-written paper about a topic that needs research. The article starts with a clearly identified and relevant research question. The inclusion and exclusion criteria are clearly written. The article also takes an effort to include people with poor literacy. The article has a strong n of 382 people. The data analysis appeared error-free.

The step taken by study designers to include two dedicated clinical workers to talk to patients and gather data was well-thought-out to tackle the under-reporting of mental health issues due to perceived stigma.

As a reviewer, this article was easy to read and understand. While there are some minor concerns, they can be remedied pretty quickly with a few minor alterations.

This article will also benefit from being reviewed by a native English speaker. As a non-native speaker, the reviewer understands the difficulty one faces writing in a language other than their mother tongue. Having the help of a native English speaker improves the readability of the article significantly.

Major concerns

The article's title talks about addressing mental health, but the article goes on to discuss common mental disorders. Mental health covers many aspects of an individual's life, and perhaps the title should be amended to say common mental disorders.

While the authors define almost all terms utilized by them. In the article, common mental disorders are not defined.

One of the ways in which the shortage of trained mental health care workers is tackled in rural America is the utilization of telepsychiatry to directly engage with the patient or providing teleconsultation from a psychiatric team to support the primary health care workers in the community. This model may have challenges being replicated in countries with poor internet infrastructure, but the interaction may be benefited from discussion of this method as well.

Outpatient departments in America also frequently give paper copies of self-help mental health screening questionnaires like PHQ 9 to patients in the waiting area after they check in/register to complete prior to them seeing their healthcare providers. This is an easy way for the patient to get their needs met and also alerts the provider to discuss this with the patient. This option could have been included in the discussion and can be considered as a solution to the quandary the writers identified in this article.

The article uses a version of AUDIT that is being validated. This should be included as a limitation.

Minor concerns

Page 7 lines 141 to 152 – these lines are single-spaced instead of double-spacing.

While the overall language of the article is very easy to understand and read, certain sections of the article read awkwardly and will benefit from restructuring. These are listed as follows

Page 4, lines 74 to 87

Page 7, lines 153 to 158

Reviewer #3: Great topic to investigate, and good data acquisition overall. Although the difference in the sample sizes might limit the quality of the data. Please revise Line 219- GP frequently exchanged?. seems like something is missing there.

Reviewer #4: Thank you for allowing me to review your study. It was an interesting read and though overall it does have interesting findings that overall there wasn't as much of a difference during the pandemic- it is also valuable to explore what might have been mitigating factors.

- I was wondering if the GPs had any data on whether these patients were Covid positive/negative. Although not comparable necessarily to previous years, it might be interesting to add.

- I'd also just like to point out some spelling typos on Page 3 Line 64- Japon instead fo Japan, Page 3 Line 67 professionnal instead of professional, Page 3 Line 69 scare instead of scarce.

- I also wonder if there is any data about what the GPs did when faced with increased spontaneously expressed SI during the pandemic if the number of suicidal attempts/ completed suicides remained generally similar and there is a general decrease in hospitalizations due to self-harm. Is that data available in the database?

- Also, was there any information about psychiatric diagnoses in these reported case? That could also be a variable to compare between pre pandemic and during pandemic?

Thank you

**********

6. PLOS authors have the option to publish the peer review history of their article (what does this mean?). If published, this will include your full peer review and any attached files.

If you choose “no”, your identity will remain anonymous but your review may still be made public.

**Do you want your identity to be public for this peer review?** For information about this choice, including consent withdrawal, please see our Privacy Policy.

Reviewer #1: **Yes: **Anil Bachu

Reviewer #2: **Yes: **Lakshit Jain MD

Reviewer #3: **Yes: **Meenal Pathak

Reviewer #4: No

**********

[NOTE: If reviewer comments were submitted as an attachment file, they will be attached to this email and accessible via the submission site. Please log into your account, locate the manuscript record, and check for the action link "View Attachments". If this link does not appear, there are no attachment files.]

While revising your submission, please upload your figure files to the Preflight Analysis and Conversion Engine (PACE) digital diagnostic tool, https://pacev2.apexcovantage.com/. PACE helps ensure that figures meet PLOS requirements. To use PACE, you must first register as a user. Registration is free. Then, login and navigate to the UPLOAD tab, where you will find detailed instructions on how to use the tool. If you encounter any issues or have any questions when using PACE, please email PLOS at figures@plos.org. Please note that Supporting Information files do not need this step.

# Author comment: Author response to Decision Letter 0

**DOI:** 10.1371/journal.pone.0278266.r002

17 Oct 2022

Point-by-point response to reviewers' 

Additional Editor Comments:

We thank a lot the Editor for having reviewed this article, and for all the interesting comments. 

ABSTRACT

1. add objectives as a separate subsection

We added the objectives as a separate subsection.

2. Line 43 limitation is not needed here

We deleted this sentence. 

3. What were the P-values for the differences in IR? Pls specify

We added the P-values for the differences in mean incidence rates during vs before the pandemic (Mann-Whitney test). 

Page 2, lines 18-21: The mean IRs were 52 (95%CI = 44; 57) per 100,000 inhabitants for SAs during the pandemic versus 47 [36; 57] during the pre-pandemic period (p = 0.49), and 5 (95%CI = 2; 9) for CSs versus 11 [6; 16] (p = 0.30).

INTRODUCTION

1. Can you elaborate more on suicidal behaviors and related consequences in France? As in the US, those are crisis situations where patients go straight to the hospital and are not managed outpatient basis. 

Thank you for your remarks on the management of suicidal behaviors by GPs that may be specific to some countries. While the crucial role of GPs in suicide prevention among patients with suicidal ideation may be universal, we recognize that the implication of the GP on crisis situations may vary according to system health organizations and culture. In France, if the majority of patients go straight to ER, some choose to see their GPs (for suicidal ideas or after a suicide attempt depending in these cases on the severity of the act). This explain why a previous study in a representative sample of the population (N=6500) showed that 39.3% of individuals did not present to hospital (53.4% in 18-24-year-old) after suicide attempts. Among them, 37.7% were reported visiting a doctor or a psychiatrist/psychologist (Jollant et al. 2020). Another study conducted by our team (Younes et al. 2020) reported that 55.4% of SA reported by GPs were directly managed by an emergency department where they go straight and 29.6% (95/321) were managed by the GPs at the time of the SA (and the GPs addressed the majority of them to the ER). GPs are central in the management at a distance from a suicidal act. These studies were conducted before the COVID-19 pandemic. Many authors have showed that the restriction measures (lockdowns) had an impact on the number of hospital consultations, and notably a decrease of mental health consultations at the beginning of the pandemic. It may be surprising since several studies (including in France) showed an increase in mental health disorders at that time. During this unprecedented time, in a country such as France, it would be possible that patients may refer more to their GPs after a suicidal act than before the pandemic. We added sentences to describe and highlight the role of GP in the moments around a suicidal act in France. We also added data on suicide attempts trends during the pandemic in the country. 

Page 2, line 4: Trends from primary care in a country such as France are crucial since persons may not consult hospital services after suicide attempts (SA) but rather see their general practitioner (GP).

Page 4, lines 9-14: Studies examining the impact of the COVID-19 pandemic on suicidal acts in general practice are scare [13, 14, 23]. General practitioners (GPs) have a central role in suicide prevention [24], which was equally true during the pandemic [25]. Moreover, in a country such as France, GPs play a crucial role in the management of patients after a SA. A study from a representative sample of the French population showed that 39.3% of individuals did not visit a hospital (53.4% of 18-24 year-olds) after a SA [26]. Among them, 37.7% reported visiting a doctor or a psychiatrist/psychologist. Another study showed that French GPs were more likely to be involved in the management of the patient at the time of the SA if the patients were younger [27]. This may have been exacerbated during the pandemic due to hospitals being overwhelmed or the fear of going to the hospital.

Page 4, lines 18-23: In France, an overall 8.5% decrease in the total number of self-harm hospitalizations was reported during the COVID-19 pandemic from September 2020 to August 2020 [16]. Its remains at lower level than expected until August 2021 [20]. On the contrary, the number of calls for intentional drug or other toxic ingestions to the French poison control centers were above what was expected during the COVID period [28]. These studies reported differences according to age and gender, with young females being particularly affected by the persistence of the pandemic. Evidence is currently lacking, but it is likely that the numbers and characteristics of SAs and CSs among primary care patients have changed during the pandemic.

Page 5, line 4: Using data from the FSN, we aimed to compare incidence rates and characteristics of SAs and CSs reported by French GPs during the two years of COVID-19 pandemic to those of the preceding ten years.

Authors have included SA/CS which is a major crisis and learning the trend in GPs does not define the overall incidence/trend of suicidality during pandemic vs. other ten years' data. 

We agree with the fact that our study do not define the overall trend of suicidality during pandemic vs. other ten years' data. We included SA/CS but not suicidal ideation or intent, which are also a part of suicidality as you rightly pointed out, because these data are not available (the scientific committee had decided to target the report of SA and SC, which are less frequent than suicidal ideas in primary care for this reason). We modified few sentences to be more precise on this point. We deleted the terms ‘suicidal behaviors’, ‘suicidal persons’ and replaced them by more specific terms: 

Page 1, lines 1-4 (Title): Trends and characteristics of attempted and completed suicides reported to general practitioners before vs during the COVID-19 pandemic in France: data from a nationwide monitoring system, 2010-2022

Page 2, lines 4-6: Trends from primary care in a country such as France are crucial, as individuals may not consult hospital services after suicide attempts (SAs) but rather see their general practitioner (GP).

Page 3, line 15: suicidal acts

Page 4, line 6: suicidal acts

Page 4, lines 23-25: Evidence is currently lacking, but it is likely that the numbers and characteristics of SAs and CSs among primary care patients have changed during the pandemic.

After reading the methods it seems this is a follow-up with patients who had SA/CS. If yes, then pls provide a brief on study design in goals as well as the title needs to be changed.

We did not use the term of ‘follow-up’ since we did not used data from a cohort population, with a stricto-sensu follow-up of persons. Indeed, in the French health system, patients can freely choose their GP. Thus, GP may follow some patients (who choose to consult the same GP during their entire life), but may also see a patient once only (if the patient then decides to consult another GP). But we used data collected by a nationwide monitoring system. It hence allowed the comparison across time of SA/CS. We added words on study design in goals and in the title.

Page 1, lines 1-4: Trends and characteristics of attempted and completed suicides reported to general practitioners before vs during the COVID-19 pandemic in France: data from a nationwide monitoring system, 2010-2022.

Page 2, lines 12-13: We conducted a retrospective observational study using data from a nationwide monitoring system, the French Sentinel Network (FSN)

Page 5, lines 1-2: Since 1999, GPs participating in the French Sentinel Network (FSN), a nationwide, near real-time monitoring system, have reported SAs and CSs occurring among their patients. 

METHODS

1. I will like the authors to clarify that this is the patient-reported history of CS/SA in goals as well as title

Thank you for your remark. We agree with you about the importance to clearly mentioned it, since Sentinel GPs report SAs and CSs whether they were reported by the patient him/herself or by other persons (whether or not they have seen the patients at the time of the act). We thus modified the title and the goal (in abstract and in the manuscript). We do not use the precise term of ‘patient-reported’ which may refer to ‘patient-reported outcome’ defined in the literature as an outcome reported by the patient (in our case, SA/CS may be reported by family/friends/hospital reports). But we used the term of “attempted and completed suicides reported to general practitioners” to take into consideration your remark. 

We also modified it in the variables’ subsection (outcome), in the discussion section (we added sentences in limits). 

Page 1, lines 1-4: Trends and characteristics of attempted and completed suicides reported to general practitioners before vs during the COVID-19 pandemic in France: data from a nationwide monitoring system, 2010-2022

Page 2, lines 8-10: We aimed to evaluate whether the incidence and characteristics of SAs and completed suicides (CSs) reported to French GPs were different during the COVID-19 pandemic than those of before

Page 2, lines 4-5: The COVID-19 pandemic had no major effect on the overall incidence of SAs and CSs reported to French GPs. However, more suicidal acts were reported among younger and older individuals.

Page 5, lines 1-2: Since 1999, GPs participating in the French Sentinel Network (FSN), a nationwide, near real-time monitoring system, have reported SAs and CSs occurring among their patients.

Page 7, lines 8-10: The outcomes of this study were the incidence rates for SAs and CSs reported to the French GPs during the COVID-19 pandemic in comparison with the pre-pandemic period.

Page 16, lines 12-13: The COVID-19 pandemic did not increase the incidence of SA and CS reported to the French GP, consistent with results of previous studies conducted in high-income and upper-middle-income

Page 19, lines 17-21: First, SAs and CSs may have been underreported by GPs. Indeed, GPs have reported to the FSN suicidal act in their patients whether they were reported by the patient him/herself or by other professional caregivers or their family. We assume that GPs may not be aware of some suicidal acts (for patients less involved in general practice). Moreover, GPs might forget to report all cases. We nevertheless found similar SAs:CSs ratios during the different periods, and to those in other general practice networks [63-65].

Page 20, lines 17-20: In conclusion, there is no major impact of the COVID-19 pandemic on the overall incidence rates of SA and CS reported to the French GPs during the first two years. However, more suicidal acts were reported among younger and older individuals. More studies including data of late 2021 and 2022 are needed to confirm these results. 

2. Subsection and more defined inclusion and exclusion criteria, variables, and outcomes need to be added. Also, summarize current content in the statistical analysis subsection and the last para under ethical approval.

Thank you for your remark. We added subheadings ‘Study design and settings’ (page 5 line 8), ‘French primary care system particularities and data sources’ (page 5 line 21), ‘Data collection, case definition and inclusion criteria’ (page 6 line 12), ‘Variables’ (page 7 line 7), ‘Statistical analysis’ (page 8 line 3) and ‘Ethical statements’ (page 8 line 25). 

We defined the inclusion and exclusion criteria as following. 

Page 7, line 4-5: All cases reported by Sentinel GP from March 11, 2010 to March 10, 2022 were included in the analysis, excepted those for whom the vital outcome of the suicidal act was missing (n=64).

We defined the variables as following. 

Page 7, lines 10-26: The COVID-19 pandemic period from March 11, 2020 (the World Health Organization declared on that day the global pandemic) to March 10, 2022. The ‘pre-pandemic period’ was defined from March 11, 2010 to March 10, 2020, as reference. Age groups were categorized according to predefined limits (≤25, 26-65, >65). Suicide methods were grouped into a single dummy ‘violent method: yes/non’ variable according to previous research (“non-violent methods” included self-poisoning by pharmacological agents ± alcohol, gases, or other toxic substances, and “violent methods” included hanging, firearms, self-cutting, jumping from a height, crashing a car, or jumping or lying in front of a train or a car) [35]. Two geographical variables were created from the zip code of the GP who reported SAs and CSs during the periods of interest. The first was a five-category variable, defined according to the telephone area code (Ile-de-France, Northwest, Northeast, Southeast, Southwest), consistent with the various degrees of pandemic intensity across the French regions as described in the ‘settings’ subsection [36]. The second was a rural/urban dummy variable was created from the zip code of the GP based on French national statistical institute (INSEE, France).

We defined the outcome as following. 

Page 7, lines 8-10: The outcomes of this study were the incidence rates for SAs and CSs reported to the French GPs during the COVID-19 pandemic in comparison with the pre-pandemic period. SAs and CSs were defined according to the vital outcome of the suicidal acts reported by the Sentinel GPs.

We summarized the content in the statistical analysis subsection. 

RESULTS

1. Table 1 pls add p-values to support the difference between both IRs. Recommend creating a table with columns between pre-pandemic and pandemic. Currently, you kept them as rows. Recommend to create a line/bar graph trend of IR from 2010 to 2022 and a linear-by-linear association test to evaluate the p-values across the trend. The flaw in analysis to compare IR between pre and during pandemic needs to be addressed

Thank you for your remark. We created a table with columns between pre-pandemic and pandemic for mean annual incidence rates of SA and CS (Table 1 on page 11). We added p-value for the differences in mean incidence rates during vs before the pandemic (Mann-Whitney test) in the table as well as in text. Detailed values (annual incidences per years were added in supplementary material). 

As you recommended, we also created a line graph trend of annual incidence rates from 2010 to 2021 (Figure 1 on page 10). Thank you for your suggestion. The curves being relatively flat, we decided after discussion with statisticians to not performed statistical test to evaluate the p-value across the trends (and also because our objective was to assess whether the incidences rates were changed during the pandemic compared to before, and not to assess the trends of the SA/CS during the 10 last years). We thus decided to present i) first the Figure 1 that you recommended, which shows flat curves for SA/CS since 2010. Indeed, all confidence intervals overlap, which confirmed that the incidence rates did not change (during the pandemic or during these last 10 years), ii) second the mean annual incidence rates of SAs and CSs (with p-value for differences). Altogether, we think these results show that incidence rates for SAs and CSs were stable during the pandemic compare to before, and also provide the readers the mean values (detailed in supplementary table if needed). 

We recognize that the comparison of mean IRs between pre- and during the pandemic has limitations (and would make no sense without the flat curves presented on Figure 1). Given the flat curve, we thought it is not necessary to perform any more complicated statistical test (trends, expected vs observed etc.). We added these points in the limitations (discussion section). 

Page 2, lines 17-21: The IRs remained stable for SAs and CSs before and during the pandemic (overlap in confidence intervals). The mean IRs were 52 (95%CI = 44; 57) per 100,000 inhabitants for SAs during the pandemic versus 47 [36; 57] during the pre-pandemic period (p = 0.49), and 5 (95%CI = 2; 9) for CSs versus 11 [6; 16] (p = 0.30).

Page 8, lines 14-18: Significance of trends was analyzed by plotting estimated annual incidence rates over years and assessing overlap of confidence intervals. We considered the incidence rates significantly different if no overlap in confidence intervals were present. Average annual incidence rates for before vs during the pandemic were compared using the Mann-Whitney test.

Page 9, lines 23-25, page 10 lines 1-3: Annual incidence rates for SA and CS between 2010-2021 are showed in Figure 1. Detailed data are provided in Table S2. Between 2010-2022, the annual incidence rates remained stable for SAs and CSs (no overlap in confidence intervals, Figure 1 and Table S2). For SA, the mean annual incidence rates were of 52 (95%CI = 44; 57) per 100,000 inhabitants during the pandemic versus 47 (95%CI = 36; 57) before the pandemic (p = 0.49). For CS, the mean annual incidence rates were of 5 (95%CI = 2; 9) versus 11 (95%CI = 6; 16; p = 0.30) (Table 1).

Page 20, lines 3-10: Fourth, suicidal acts are rare events in a GP’s practice population, and small sample sizes may lead to low statistical power for the detection of differences between periods. This also made not possible the study of trends according to smaller periods or/and to age and sex. Moreover, we recognized that the comparison of mean annual IRs has very limited value. However, given the flat curve of trends for SA/CS incidence rates, we believed that it was not necessary to perform more complicated statistical models to show that the incidence rates were stable during the pandemic compare to before. Again, our small sample size would have limited the use of such models.

2. Good work on table 2. Why there are two N's. I assume it shows total data collected for that variable and if you reporting missing data then pls only include "n" in the table and calculate missing data from "N" and report it as footer line.

Thank you for your suggestions. Yes, the second N showed the total data collected for that variable. Changed were made. 

3. Table 2 and 3 can be merged

Since Table 2 presents comparison between characteristics during the pandemic versus before, and table 3 presents comparison between the first year versus the second year of the pandemic, we decided to not merge this different tables. 

DISCUSSION

1. I would like to see data results from either France/ European studies related to similar goals and compare the findings, and if there is inpatient study on IR and related factors on suicide then pls compare the findings.

Page 16, lines 12-27, page 17, lines 1-20: 

The COVID-19 pandemic did not increase the incidence of SA and CS reported to the French GP, consistent with results of previous studies conducted in high-income and upper-middle-income [37]. During the initial phase of the pandemic, stable or decrease incidence for CS were also reported in Norway [6], Finland [38], England [39], Ireland [40], Germany [41], Greece [42], Sweden [43] and Austria [44]. Similar results were showed for self-harm presentations to hospitals in the United-Kingdom [45], Spain [46], Portugal [47], France [16], from primary care in the UK [13, 14], and from poison control centers in France [20]. Thus, studies conducted with data from different sources, i.e. hospital, primary care, and poison control centers, suggest a true decrease in the number of suicidal acts during the first part of the pandemic. The absence of an increase in suicidal acts during the pandemic could be explained by better social support, financial help provided by governments, and reduced stress related to school or work. Although we found stable SA and CS rates, it is possible that trends may vary according to age and sex groups. In Japan and France, a decrease of SAs or CSs during the first months of the pandemic followed by an increase in young females has been reported [7, 20]. Similar results were reported from the French poison control center data [28]. Studies conducted by Public Health France in a representative sample of the French adult general population reported high rates of depression, anxiety, sleep problems, and suicidal ideas during the first two years of the pandemic at levels superior to the pre-COVID period. These results highlight the importance of monitoring depressive/anxiety symptoms, and suicidal ideation, in particular in primary care.

In line with previous studies, our data suggests higher risk of SA among young/students or old people, and among students and retirees during the pandemic compared to before [13, 14, 16, 19, 22, 48]. In France, an increase in hospitalization for self-harm was found in adolescent girls, notably between January 2021 and August 2021, while middle-aged adults showed a decrease [20]. An increase in calls to the poison control center from mid-2020 to May 2020 was also observed in young females, and in in older-aged people [28]. Young and students were particularly affected by the unusual school calendar during the pandemic [7, 49] and significant disruptions in social environment [50, 51]. Older people were more concerned by severe COVID-19, and restrictions in visits and travel particularly favored social isolation for this group, especially for those living alone [52, 53]. While the unemployment risk factor may have been somewhat mitigated by better financial and social support, whether the pandemic have reduced work-induced stress for certain people should be elucidated. 

2. Conclusion needs to be revised after re-analysis as recommended. A very strong statement is made and needs to be rectified.

Thank you for your remark. We modified the conclusion. 

Page 20, lines 17-21: In conclusion, there is no major impact of the COVID-19 pandemic on the overall incidence rates of SA and CS reported to the French GPs during the first two years. However, more suicidal acts were reported among younger and older individuals. More studies including data of late 2021 and 2022 are needed to confirm these results. 

Reviewer 1

I believe the research question, and aim of the study has been clearly outlined. The methods in the study design are appropriate to answer the aim. The variables are clearly defined. The results are stated clearly, the date presented in a clear and appropriate way. The conclusion answers the aims of the study. the references seem relevant. I am not aware of any key studies were missed that has been referenced.

We thank you for your review and for your interest in our article.

Reviewer 2 

This is a well conceptualized, well thought out and well performed study on COVID-19 and its impact on mental health, specifically suicide. Following the outbreak of COVID-19, many publications relaxed their criteria’s and began publishing a lot of work on COVID-19. While this has led to a significant amount of literature being available, a lot of it is hastily written, focuses on the individual's experience during the early part of COVID-19 and unfortunately does not provide overall long-term opinion of how COVID-19 has impacted the mental health of people. Long-term studies over the impact of COVID-19 are desperately needed in order to quantify the learnings from the onset of this pandemic and how the society reacted to it so that one can plan for the next pandemic. 

This study also examines the important role general practitioners play in managing mental health, which is often under-appreciated and sometimes even derided by contemporary psychiatrists.

We thank you for your review and for your interest in our article. We actually took the time to set up a study that was complementary to existing and rapidly published data, making sure to study the deferral of suicidal acts over a long period of time (up to March 22), and comparing it to the previous 10-year period. We too are convinced of the important role of GPs. We think this type of data can help remind everyone. 

As a reviewer, this article was easy to read and understand for the most part. While there are some minor concerns, they can be remedied quickly with a few minor alterations.

This article will also benefit from being reviewed by a native English speaker. As a non-native speaker, the reviewer understands the difficulty one faces writing in a language other than their mother tongue. Having the help of a native English speaker improves the readability of the article significantly.

Following your suggestion, the revised version of the manuscript has been reviewed by a native English speaker. Several changes have been made.

Major concerns

This article's introduction can be improved by a discussion of the French sentinel network, specifically the number of people that the network covers so that one can quantify the importance of this network. This is also relevant at Page 4 lines 84-95, where the discussion will be benefited by hard numbers (Rather than percentage of population) to allow the reader to conceptualize the number of people that this article is dealing with.

Thank you for this comment. Around 500 GP participate to the surveillance of the French sentinel network (FSN) each week (1.2% of the French GP population). However, we are unfortunately not able to estimate the number of French patients covered by the FSN. Indeed, in France, GP do not serve a defined practice population: all residents may freely choose their GP (in contrast to systems with a stricter registration with a GP). We could make the hypothesis that Sentinel GP covers 1.2% of the French population (more than 800,000 hab). Since we don’t have any official data about this number, we prefer to not mention it in the article. This is also the reason why we did not improve the introduction by adding the number of people the French sentinel network covers. 

However, created and reorganized the ‘French primary care system particularities and data source’ subsection. We added sentences about the French primary care particularities. 

We also indicated that the FSN is nationwide in the Title and in the text. 

Page 5, line 24: France provides universal health insurance for its population under a system that reimburses GPs on the basis of a national fee schedule. This primary healthcare system offers coverage for all residents across the country. GPs do not serve a defined practice population: primary care offers coverage for all residents, and all residents may freely choose their GP. Since 1984, the FSN, a nationwide monitoring system, has collected near real-time epidemiological data from participating French GPs. For the purpose of this study, we used data on SAs and CSs reported by GPs to the FSN.

The FSN comprises approximately 500 voluntary Sentinel GPs (1.2% of the French GP population) who routinely report data on health indicators, including suicidal acts.

Page 1, lines 1-4 (Title): Trends and characteristics of attempted and completed suicides reported to general practitioners before vs during the COVID-19 pandemic in France: data from a nationwide monitoring system, 2010-2022

Page 2, lines 12-13: We conducted a retrospective observational study using data from a nationwide monitoring system, the French Sentinel Network (FSN).

Page 5, lines 1-2: Since 1999, GPs participating in the French Sentinel Network (FSN), a nationwide, near real-time monitoring system, have reported SAs and CSs occurring among their patients.

As this article will be read by people who are not knowledgeable of the differences among different parts of France, the authors can discuss what makes, for example, Northwest of France different from Southwest to France in terms of industrial development, regional economy, mean income, and other parameters. While this is partially addressed in page 12, lines 247 to line 252, a demographic discussion is useful to quantify risk factors that lead to poor mental health.

We thank you for this remark. We agree with the fact that we should give to the readers more elements about the differences among different parts of France to have a full interpretation of our results. 

We mainly choose this geographical split since it was in line with the level of contamination to the SARS-CoV-2 during the pandemic (at the end of the first lockdown period, the Île-de-France and North-East had the highest reported cumulated rates of hospitalization for or deaths from COVID-19, and the South-Est had a low reported rate; during the second year of the pandemic, the South-East were the most impacted region). 

As you rightly point out, the French population's standard of living is not consistent throughout the country. According to the French National Institute for Statistics and Economic Studies, the poorest administrative departments in metropolitan France (with the highest employment rates and lowest income levels) are in the north and on part of the Mediterranean coast in the south during the last decade (INSEE, France). As of 2017, 43% of those belonging to a very high-income French household, i.e. the wealthiest 1% in the country, resided in Île-de-France, a region immediately surrounding the capital of France, Paris.

Our data on the distribution of SA/CS according to geographical areas during the pandemic versus the pre-pandemic period are difficult to interpret since the distribution of participating Sentinel GPs also changed during the pandemic compared to before (cf. Table S2). This is mentioned in the limits paragraph in the discussion section. Thus, we preferred to not discuss these differences. We added some sentences in this sense in the discussion. We also added sentences in the ‘study design and settings’ subsection. 

Page 5, lines 18-21: While the lockdowns were nationwide, the level of contamination and deaths varied greatly between administrative regions: during the early part of the pandemic, the Île-de-France and the North-East had the highest reported cumulative rates of hospitalization or death from COVID-19, and the South-Est had a low reported rate. 

Page 18, lines 8-23: The geographical difference we found for SA between the first and the second year of the pandemic is consistent with the regions where the level of SARS-CoV-2 contamination was higher (first year: Northeast; second year: Southeast)[56]. In view of a previous study that reported a weakly and negatively correlation between self-harm hospitalizations and COVID-19 hospitalizations across France, our results suggest that in region highly affected by the COVID-19, patients may have been seen in primary care instead of hospital. However, we did not find difference for Ile-de-France, whereas it was one of the regions with the highest reported cumulative rates of hospitalization or death from COVID-19 during the early part of the pandemic. It may be explained by the variation of the French population's standard of living throughout the country. Indeed, poorest administrative departments are located in the north and on part of the Mediterranean coast, while 43% of those belonging to a very high-income French household, i.e. the wealthiest 1% in the country, resided in Île-de-France, a region immediately surrounding the capital of France, Paris. More studies are needed to explore the impact of the COVID-19 pandemic on suicidal acts according to different socio-economic levels. We also found a geographical difference for SA between the pre-pandemic period and the pandemic. However, our sample of participating GPs were not comparable before vs during the pandemic, leading to difficult interpretation.

Many of the risk factors that lead to infection of and complications of COVID-19 are also predictors of poor mental health, for example poor nutrition, lower socioeconomic status etc. While a discussion of these risk factors may be out of scope of this study, these are relevant issues to think of in future research.

Thank you for this interesting remark. 

Minor concerns 

While the overall language of the article is very easy to understand and read, certain sections of the article read awkwardly and will benefit from restructuring. Some of these are listed as follows

Page 3, lines 56 to 57 

Page 4, lines 99 to 100 

Page 11 line 227 

Page 12 line 243

We modified the mentioned sentences. Based on all reviewer’s comments, this article was reviewed by a native English speaker to improve its readability. Several changes have been made.

Japan is misspelled at page 3 line 64 

Scarce is misspelled at page 3 line 69 

Sentinel is misspelled at page 11 line 215 and page 13 line 283

We modified the mentioned words.

On page 3, line 75 "It remains to be clarified"- this sentence is unnecessary."

We deleted this sentence. 

Reviewer 3

Great topic to investigate, and good data acquisition overall. Although the difference in the sample sizes might limit the quality of the data. Please revise Line 219- GP frequently exchanged?. seems like something is missing there.

Thank you for your comments. We reformulated the sentence. 

Reviewer 4

Thank you for allowing me to review your study. It was an interesting read and though overall it does have interesting findings that overall there wasn't as much of a difference during the pandemic- it is also valuable to explore what might have been mitigating factors.

- I was wondering if the GPs had any data on whether these patients were Covid positive/negative. Although not comparable necessarily to previous years, it might be interesting to add.

Thank you for your comments. We agree with your remark on the importance of having data on the history of COVID-19 among patients with suicidal behaviors. We did not have the information whether patients were Covid positive/negative. We partially collected this information thorough the following questions addressed to the GP: ‘In your opinion, is the SA/CS at least partially linked with the COVID-19 pandemic?’ Answer: yes/no. ‘If yes, is the SA/CS at least partially linked with the following situations?’ Multiple answers allowed: social isolation/family stress/fear of the COVID-19/material and financial consequences/COVID-19 disease/loss of a loved-one to COVID-19. Since these data were not comparable to previous years, we compared their distribution during the first year of the pandemic (when more stringent measures were applied to contain the SARS-CoV-2) to those of the second year. Results (only one variable which regrouped the two modalities ‘COVID-19 disease/loss of a loved-one to COVID-19’) are presented on Table 3 (for those statistically significant) and on Table S4 (all details). 

We added in ‘Results’ and ‘Discussion’ sentences to highlight these results and we added it in limits.

Page 12, lines 16-18: According to GP, 15.9% of SA and 31.1% of CS had at least partially a link with the COVID-19 pandemic. Among them, the most frequent COVID-related factor was social isolation for both SA (67.7%) and CS (71.4%). 

Page 19, lines 8-14: We found that were identified by the GP as influencing self-harm in only 15.9% of suicide attempters and 31.1% of suicide completers, COVID-19 pandemic, which is less than reported in a previous study conducted in England among adults (46.9%) [51] or in the United-States among adolescents (47.2%) [11]. However, the first study was conducted during the first lockdown, when closure of services and isolation were at its maximum level, and the second among adolescents, particularly concerned by social restrictions. Regarding COVID-19, loneliness was far ahead the most frequent evocated reasons, in line with previous studies [52]. 

Page 20, lines 14-15: Finally, we did not collect information on COVID-19 status. 

- I'd also just like to point out some spelling typos on Page 3 Line 64- Japon instead fo Japan, Page 3 Line 67 professionnal instead of professional, Page 3 Line 69 scare instead of scarce.

Thank you for your review. We modified the mentioned words. Based on all reviewer’s comments, this article was reviewed by a native English speaker to improve its readability. Several changes have been made.

- I also wonder if there is any data about what the GPs did when faced with increased spontaneously expressed SI during the pandemic if the number of suicidal attempts/ completed suicides remained generally similar and there is a general decrease in hospitalizations due to self-harm. Is that data available in the database?

Unfortunately, we do not have data on patient management by GPs after SI expression during the pandemic. We collected just information on patient management after the SA in 2013-2016 in line with our research interest at that time (published work: Younes et al. 2020). Given the disruptions in patient mental health care at hospital reported during the pandemic (first wave in particular) in many countries including France, we recognized that having collected this information during the pandemic would have been interesting to assess the impact of the pandemic on patient with SI management by GP. We added it in the limitations. 

Each year, we review the questionnaire for reported SA/CS (since it can evolve according to our research interest and context). We will add your interesting remark on SI management by GP to our next discussions. 

Page 20, lines 11-14: Sixth, we could not examine the impact of the pandemic on psychosocial factors, on psychiatric diagnoses (as information was not collected before 2020), and on the management of SI expression or of SAs/CSs (as information has not collected since 2017).

- Also, was there any information about psychiatric diagnoses in these reported case? That could also be a variable to compare between pre pandemic and during pandemic?

Yes, there was information about psychiatric diagnoses in the SA/CS reported cases. Unfortunately, these data were not collected before the pandemic (more precisely, data collected before the pandemic were about the history of mental health disorders during the last year, which is not directly comparable with data collected during the pandemic ‘Did the patient had psychiatric troubles? If yes, which one?). That is why we only compared these characteristics during the first year of the pandemic to the second year, as reported under the table 3 (not presented as non-significant), and as detailed in Table S4.

We modify the ‘Results’ and ‘Discussion’ section to added the descriptive data we had on psychiatric diagnoses during the pandemic, and discussed them. 

Page 12, lines 13-16: GP identified psychiatric disorders for 69.6% and 71.2% of SA and SC, respectively. The most frequent psychiatric disorder among both SA and CS was depression or mood disorders (77.5% and 73.0% respectively), followed by anxiety (33.8% and 35.1%, respectively) and substance use disorders (25.8% and 18.9%, respectively).

Page 19, lines 3-8: The clinical characteristics of suicidal acts (psychiatric disorders, stressors) during the COVID pandemic are similar to their already known distribution before the pandemic, while the COVID-related stressors are not really frequent in our sample [49]. We found that history of mental health disorders (particularly depression) was the most frequent factors, in line with other studies conducted during the pandemic [50], and before. Psychiatric disorders are the highest risk factor for SA/S, especially depression [49].

We agree with the fact that it is an interesting element to discuss since the pandemic may have modified the characteristics of SA or CS in terms of history of psychiatric troubles. We added it in limitations. 

Page 20, lines 11-14: Sixth, we could not examine the impact of the pandemic on psychosocial factors, on psychiatric diagnoses (as information was not collected before 2020), and on the management of SI expression or of SAs/CSs (as information has not collected since 2017).

Thank you for your remarks.

Attachment Submitted filename: Response to Reviewers.docx Click here for additional data file.

# Editor report: Decision Letter 1

**DOI:** 10.1371/journal.pone.0278266.r003

**Authors:** Rikinkumar S. Patel

14 Nov 2022

Trends and characteristics of attempted and completed suicides reported to general practitioners before vs during the COVID-19 pandemic in France: data from a nationwide monitoring system, 2010-2022

PONE-D-22-18701R1

Dear Dr. Pouquet,

We’re pleased to inform you that your manuscript has been judged scientifically suitable for publication and will be formally accepted for publication once it meets all outstanding technical requirements.

Within one week, you’ll receive an e-mail detailing the required amendments. When these have been addressed, you’ll receive a formal acceptance letter and your manuscript will be scheduled for publication.

An invoice for payment will follow shortly after the formal acceptance. To ensure an efficient process, please log into Editorial Manager at http://www.editorialmanager.com/pone/, click the 'Update My Information' link at the top of the page, and double check that your user information is up-to-date. If you have any billing related questions, please contact our Author Billing department directly at authorbilling@plos.org.

If your institution or institutions have a press office, please notify them about your upcoming paper to help maximize its impact. If they’ll be preparing press materials, please inform our press team as soon as possible -- no later than 48 hours after receiving the formal acceptance. Your manuscript will remain under strict press embargo until 2 pm Eastern Time on the date of publication. For more information, please contact onepress@plos.org.

Kind regards,

Rikinkumar S. Patel, M.D., M.P.H.

Academic Editor

PLOS ONE

Additional Editor Comments (optional):

Thank you for the great hard work done by all the authors and addressing the recommendations made by the reviewers. Your project is well-designed and conducted with appropriate manuscript drafting that will gain attention of interested readers.

# Editor report: Acceptance letter

**DOI:** 10.1371/journal.pone.0278266.r004

**Authors:** Rikinkumar S. Patel

6 Dec 2022

PONE-D-22-18701R1 

Trends and characteristics of attempted and completed suicides reported to general practitioners before vs during the COVID-19 pandemic in France: data from a nationwide monitoring system, 2010-2022 

Dear Dr. Pouquet:

I'm pleased to inform you that your manuscript has been deemed suitable for publication in PLOS ONE. Congratulations! Your manuscript is now with our production department. 

If your institution or institutions have a press office, please let them know about your upcoming paper now to help maximize its impact. If they'll be preparing press materials, please inform our press team within the next 48 hours. Your manuscript will remain under strict press embargo until 2 pm Eastern Time on the date of publication. For more information please contact onepress@plos.org.

If we can help with anything else, please email us at plosone@plos.org. 

Thank you for submitting your work to PLOS ONE and supporting open access. 

Kind regards, 

PLOS ONE Editorial Office Staff

on behalf of

Dr. Rikinkumar S. Patel 

Academic Editor

PLOS ONE
